# Cardiopulmonary arrest and cardiopulmonary resuscitation in pregnancy Number 3 – 2026

**DOI:** 10.61622/rbgo/2026FPS3

**Published:** 2026-04-16

**Authors:** Álvaro Luiz Lage Alves, Lucas Barbosa da Silva, Roxana Knobel

**Affiliations:** Hospital das Clínicas Universidade Federal de Minas Gerais Belo Horizonte MG Brazil Hospital das Clínicas, Universidade Federal de Minas Gerais, Belo Horizonte, MG, Brazil.; Hospital das Clínicas Universidade Federal de Minas Gerais Belo Horizonte MG Brazil Hospital das Clínicas, Universidade Federal de Minas Gerais, Belo Horizonte, MG, Brazil; Faculdade de Medicina Universidade Federal de Santa Catarina Florianópolis SC Brazil Faculdade de Medicina, Universidade Federal de Santa Catarina, Florianópolis, SC, Brazil.

The National Specialized Commission on Obstetric Emergencies of the Brazilian Federation of Gynecology and Obstetrics Associations (Febrasgo) endorses this document. The content is based on scientific evidence on the proposed topic, and the results presented contribute to clinical practice.

## Key points

Early diagnosis of cardiopulmonary arrest during pregnancy is directly related to the success of cardiopulmonary resuscitation.
Treatment of cardiopulmonary arrest during pregnancy requires a trained team, although resuscitation maneuvers should be initiated by the professional who made the diagnosis. The approach should be multidisciplinary, rapid and led by a professional with knowledge of cardiopulmonary resuscitation and its specificities in obstetric patients. Ideally, the assistance should also include an anesthesiologist or intensivist with skills in difficult intubation, an obstetrician or surgeon to perform resuscitative hysterotomy (perimortem cesarean section), nursing professionals with experience in obtaining venous access, preparing and administering medications, and a neonatology team.
The reversible causes of cardiopulmonary arrest during pregnancy are summarized by the acronym of the 6Hs and 6Ts: hypoxia, hypovolemia, hydrogen ion (acidosis), hypo/hyperkalemia, hypothermia, and hypo/hyperglycemia; and by toxins, tamponade (cardiac), tension pneumothorax, thrombosis (pulmonary), thrombosis (coronary), and tablets (drug poisoning).
Most cardiopulmonary arrests during pregnancy occur due to non-shockable rhythms (pulseless electrical activity and asystole), which are resolved through high-quality cardiopulmonary resuscitation and correction of reversible causes.
Ventricular fibrillation and pulseless ventricular tachycardia are the main rhythms classified as shockable.
In cardiopulmonary arrest in pregnant women, endotracheal intubation is more difficult, must be performed early, and requires a qualified professional. In the event of intubation failure or the resuscitator’s inexperience, the use of supraglottic devices is an efficient alternative.
Determining gestational age in the care of pregnant women in cardiopulmonary arrest is essential, since the gravid uterus above the umbilicus exerts aortocaval compression, compromising the success of cardiopulmonary resuscitation. Furthermore, neonatal viability, from 22-24 weeks onwards, is another important factor in the decision to perform a resuscitative hysterotomy.
The probability of a normal neonatal neurological outcome is significantly higher when resuscitative hysterotomy is completed within five minutes.
Transesophageal echocardiography and limited cardiopulmonary ultrasound can aid in the etiological diagnosis of hemodynamic collapse occurring during labor or delivery.
Pregnant women with cardiovascular collapse due to amniotic fluid embolism, pulseless electrical activity, anesthetic toxicity, cocaine intoxication, and pulmonary embolism may benefit from intra-aortic balloon pump, cardiopulmonary bypass, and extracorporeal membrane oxygenation.

## Recommendations

If the pregnant woman is unconscious, the first action should be to assess her responsiveness, verbally or physically. If there is no response, breathing and circulation (carotid) should be checked immediately.
Assessments of fetal heart rate and/or well-being are not recommended during maternal cardiopulmonary resuscitation, as the pregnant woman’s condition should guide the care.
Chest compressions should be performed with the pregnant woman in the supine position. The compression rate should be between 100 and 120 per minute, and the chest should be compressed to a depth of 5 to 6 cm.
During pregnancy, intubation should be performed with a smaller endotracheal tube (6 or 7 mm). Before intubation, two ventilations are recommended for every 30 chest compressions. After intubation, ventilations should be uninterrupted, at a rate of 8 to 10 per minute.
In cardiopulmonary resuscitation of pregnant women, venous access should be established above the diaphragm.
In pregnancies where the fundus reaches the umbilicus, manual uterine displacement is required during cardiopulmonary resuscitation. From the 20^th^ week of gestation, resuscitative hysterotomy is indicated if spontaneous circulation does not return within four minutes of arrest. This procedure should be performed within one minute at the site of collapse. While the abdominal incision may be vertical or transverse, the uterine incision must be a classic corporeal midline. Following the removal of the fetus, the uterine cavity should be packed with surgical compresses. Placental removal, hysterorrhaphy, and laparorrhaphy are deferred until maternal stabilization.
In non-shockable rhythms, epinephrine is recommended from the beginning of cardiopulmonary resuscitation, every three to five minutes. In shockable rhythms that do not respond to cardiac compressions, defibrillation and epinephrine, the administration of amiodarone is recommended. All intravenous medications should be injected as an intravenous bolus, followed by 20 mL of physiological saline solution or distilled water, and elevation of the limb.
Defibrillation in pregnant women should be performed using a biphasic shock of 120 to 200 joules. When using a monophasic defibrillator, 360 joules are recommended.
Once hemodynamic collapse has been reversed and chest compressions are no longer necessary, the pregnant woman should be positioned in the left lateral decubitus position. Hyperthermia should be avoided.
Pregnant and postpartum women with suspected cardiopulmonary arrest due to opioid use should be treated with their antagonists (e.g., naloxone).

### Background

Cardiopulmonary arrest (CPA) is defined as the interruption of the heart’s mechanical activity and confirmed by the absence of signs of circulation. Although CPA is a rare event in pregnancy, it affects two patients: the mother and the fetus. Treatment of CPA in pregnancy requires a rapid multidisciplinary approach, including a professional who leads resuscitation and has knowledge of cardiopulmonary resuscitation (CPR) and its specificities in obstetric patients. Ideally, care should also include an anesthesiologist or intensivist with skills in difficult intubation, an obstetrician or surgeon to perform resuscitative hysterotomy (perimortem cesarean section), nursing professionals experienced in obtaining venous access, preparing and administering medications, and a neonatology team.^(1)^

Effective CPR is critical for achieving return of spontaneous circulation and improving survival rates. While standard life support algorithms apply, the unique physiological and anatomical changes of pregnancy necessitate specific modifications to these protocols. The treatment of CPA during pregnancy lacks robust scientific evidence, since most studies exclude pregnant women and randomized clinical trials related to the topic are lacking. Therefore, the currently proposed approach is based on expert opinion and data from small case series and cohort studies involving patients with CPA during cesarean sections. Due to the rarity of the event and the inherent stress of the situation, specific training with simulations involving the multidisciplinary team and the standardization of procedures are very important.^(1)^

### What are the terminologies, incidence, etiology, and pathophysiology of cardiopulmonary arrest in pregnancy?

Cardiopulmonary arrest is the sudden cessation of cardiac activity in which the patient becomes unresponsive, without breathing, and without signs of circulation. This event may be categorized as fatal or non-fatal, depending on the outcome. The arrest is considered non-fatal if circulation is successfully restored, whether through medical intervention (e.g., defibrillation) or spontaneous reversion. In the absence of restoration of circulation, CPA progresses to death, and is formally termed sudden cardiac death.^(2)^

Cardiopulmonary arrest in parturient women is a rare event (1 in 9,000 hospitalizations for childbirth in the United States), but has higher probability among pregnant women of older age groups, Black or African-American women, and those with comorbidities (chronic hypertension, diabetes, smoking, asthma, nephropathy, heart disease, and mental disorders).^(3)^ A high prevalence of overweight/obesity (>60%) and anesthetic complications (24%) is also reported among pregnant women who developed CPA.^(4)^ Although Brazilian data on the etiology of CPA are nonexistent, hypertensive syndromes, hemorrhages, and infections are known to be the main causes of maternal death in the country.^(5)^ Currently, acute respiratory distress syndrome and mechanical ventilation are, respectively, the diagnosis and intervention most commonly associated with CPA.^(4)^

Cardiopulmonary arrest in pregnancy arises from either direct obstetric causes (conditions unique to pregnancy) or indirect causes (pre-existing or co-incidental conditions exacerbated by the pregnancy state). Causes of CPA in pregnancy include cardiac and non-cardiac etiologies. Among the non-cardiac causes, the following stand out: obstetric hemorrhage, sepsis, anesthetic and drug complications (such as magnesium toxicity), massive pulmonary embolism, vascular collapse (anaphylaxis, amniotic fluid embolism), stroke, trauma, and thromboembolic complications. Cardiac causes include peripartum cardiomyopathy, acute myocardial infarction (AMI), pre-existing heart disease (congenital, acquired, cardiomyopathy), malignant arrhythmias, and genetic syndromes such as Marfan. The risk is especially high in pregnant women with moderate or complex congenital heart disease, particularly cyanotic heart disease, severe pulmonary arterial hypertension, complex heart disease with sequelae, malignant arrhythmias, and Marfan syndrome.^(1,6,7)^

Classifying CPA as being of cardiac origin is recommended when non-cardiac causes are unlikely. The generic term sudden cardiac death can be used for fatal events of cardiac or non-cardiac origin that result in death. Rapid identification of the etiology is fundamental to guiding treatment, and the physiological changes of pregnancy should be considered in the approach, with special attention to obstetric and cardiovascular causes.^(1,8)^

The A to H acronym, proposed by the American Heart Association (Chart 1), is useful for memorizing the main causes of CPA to be considered in pregnancy.^(9)^ The acronym of the 6Hs (hypoxia, hypovolemia, hydrogen ion – acidosis, hypo or hyperkalemia, hypothermia, hypo or hyperglycemia) and 6Ts (toxins, tamponade – cardiac –, tension – tension pneumothorax –, coronary thrombosis – infarction –, pulmonary thrombosis, tablets – drug intoxications), proposed by the Pan American Health Organization (Chart 2), emphasizes the reversible causes of CPA.^(10)^

**Chart 1:** Most common causes of cardiopulmonary arrest in pregnant women

Letter	Cause	Etiology
A	Anesthesia (anesthetic complications)	High block Hypotension Obstructed airway Respiratory depression Local anesthetic toxicity
	Accidents (trauma)	Trauma Suicide
B	Bleeding	Coagulopathy Uterine atony Placenta accreta spectrum disorder Placental abruption Placenta previa Placental retention Uterine rupture Surgeries Transfusion reaction
C	Cardiovascular	Acute myocardial infarction Aortic dissection Cardiomyopathy Arrhythmias Valvular heart disease Congenital heart disease
D	Drugs	Oxytocin Magnesium sulfate Drug dosage error Illicit drug use Opioids Insulin Anaphylaxis
E	Embolism	Amniotic fluid embolism Pulmonary embolism Cerebrovascular accident
F	Fever	Infections Sepsis
G	General	6 H — hypoxia, hypovolemia, hydrogen ion (acidosis), hypo or hyperkalemia, hypothermia, hypo or hyperglycemia 6 T — toxins, tamponade (cardiac), tension (tension pneumothorax), coronary thrombosis (infarction), pulmonary thrombosis, tablets (drug poisoning)
H	Hypertension	Preeclampsia Eclampsia HELLP syndrome

Source: Adapted from Jeejeebhoy et al. (2015)^(^^9^^)^ and the Pan American Health Organization (2024).^(^^10^^)^

**Chart 2:** Main reversible causes of cardiopulmonary arrest in pregnant women

.	
**6H**	
Hypovolemia	Bleeding (obstetric or other, may be occult), hypovolemia related to spinal block, neurogenic or septic
Hypoxia	Cardiac events (peripartum cardiomyopathy, myocardial infarction, aortic dissection, large vessel aneurysm, etc.)
Hypo/hyperkalemia and hyponatremia	Potassium disorders associated with sepsis or thrombotic microangiopathy with renal failure, hyponatremia caused by oxytocin use
Hyper/hypoglycemia	Fatty liver of pregnancy, diabetic ketoacidosis
Hydrogen (acidosis)	Sepsis, hemorrhagic shock, severe pre-eclampsia
Hypothermia	Hemorrhagic shock
**6T**	
Thrombosis (pulmonary)	Amniotic fluid embolism, pulmonary embolism, gas embolism
Thrombosis (coronary)	Coronary artery disease, myocardial infarction
Toxin, drug intoxication	Magnesium sulfate poisoning, beta-mimetics, local anesthetics
Tamponade (cardiac)	Autoimmune diseases, chest trauma
Tension (pneumothorax)	Central venous access, chest trauma
Trauma	Blunt or penetrating trauma

Source: Adapted from Pan American Health Organization (2024).^(^^10^^)^

### How should cardiopulmonary arrest be diagnosed in pregnancy?

**Figure 1:**
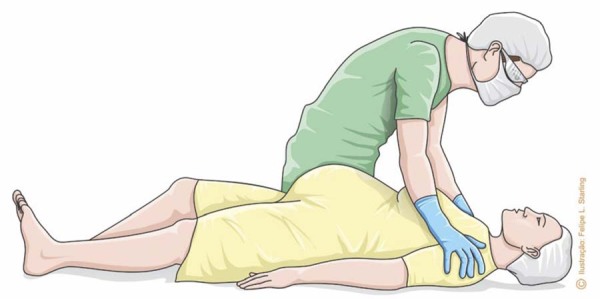
Technique for assessing the degree of verbal and physical responsiveness (trapezius squeeze)

Source: Illustration by Felipe Lage Starling (authorized).

Early diagnosis of CPA is directly related to the success of CPR. If the pregnant woman is unconscious, the first action should be to assess her responsiveness, verbally (“Are you okay?”) or physically (trapezius squeeze) (Figure 1). If there is no response, breathing and circulation should be checked immediately. Circulation should preferably be assessed by the central (carotid) pulse. Therefore, this assessment and diagnosis are potentially performed within 10 seconds. If there is any doubt, the patient will be considered apneic or with agonal breathing and no pulse.^(8)^ Since diagnostic sequencing will be the start of CPR, caregivers should pay attention to the use of personal protective equipment, preferably an N95 mask, face shield, waterproof apron, long-sleeved disposable gloves and protective eyewear.^(8,11)^

### How should the initial management and cardiopulmonary resuscitation be performed in cardiopulmonary arrest during pregnancy?

Cardiopulmonary arrest requires immediate and coordinated action. Despite the involvement of two patients, maternal resuscitation should always be the priority. Cardiopulmonary arrest and electrical cardioversion protocols for obstetrics follow the same recommendations as for non-pregnant patients. It is important to emphasize that more than 70% of CPAs in pregnancy occur due to asystole or pulseless electrical activity, which are non-shockable rhythms that can be resolved through high-quality CPR combined with correction of reversible causes.^(1)^

At the beginning of CPR, the following maneuvers and interventions should be performed simultaneously:

Calling the “maternal” code blue, provided by a multidisciplinary team (adult resuscitation in a hospital setting: anesthesiologist, obstetrician and neonatologist, nurses, nursing technicians).
Maternal positioning in dorsal decubitus on a rigid board with the head slightly tilted (Trendelenburg) and the lower limbs elevated, facilitating venous return.
Perform manual left uterine displacement if the fundus is at or above the umbilicus to relieve aortocaval decompression.
Removal of electronic fetal monitoring, if it is being performed. Fetal assessment should not be performed because, in addition to the devices hindering resuscitation maneuvers, fetal vitality will not modify the course of action.
Deliver high-quality chest compressions at a rate of 100-120 bpm and a depth of 5-6 with each movement. For patients without an advanced airway and with the uterine fundus below the umbilicus, maintain a 30:2 compression-ventilation ratio (two 350-500 mL ventilations for every 30 compressions). Provide tidal volumes of 500-600 mL (sufficient for visible chest rise); if the uterus is above the umbilicus.
Do not delay usual measures, such as defibrillation and administration of medications, when indicated.
Provide supradiaphragmatic venous access.
Assess and detect patients with difficult airways.
Estimate gestational age by measuring uterine height, aiming to identify uteri above the umbilicus or gestational age equal to or greater than 20-24 weeks.
Monitor the expired CO_2_ (capnography) when possible, in order to detect the return of circulation without the need to interrupt CPR to check the pulse (expired CO_2_ > 10 mmHg correlates with return of circulation).
Timing, notifying the rest of the team when four minutes of CPA have elapsed in order to perform resuscitative hysterotomy in pregnancies with viable fetuses.
Simultaneous treatment of causal or adjuvant factors for CPA (hemorrhage, disseminated intravascular coagulation, hydroelectrolytic disorders, cardiac tamponade, hypothermia, hypovolemia, hypoxia, hypermagnesemia, acute myocardial infarction, poisoning, pulmonary embolism, amniotic fluid embolism, anaphylaxis, tension pneumothorax, anesthetic complications, aortic dissection).^(1,12)^

### How should airway management and ventilation be performed in cardiopulmonary resuscitation during pregnancy?

Airway management should be a priority. While preparing equipment for intubation, airway patency must be ensured by extending the head and positioning a Guedel airway (temporary airway). In advanced pregnancy, both intubation and bag-valve-mask ventilation are more difficult due to physiological edema, airway narrowing, and reduced chest compliance. Bag-valve-mask ventilation with 100% O_2_ (8 to 10 breaths/minute, with both hands, > 15 liters per minute) combined with airway suctioning is the fastest, but least efficient strategy in pregnancy. It should be used while preparing the scene for endotracheal intubation, preventing desaturation. Endotracheal intubation should be performed early and requires a qualified professional. In the absence of this, prolonged attempts should be avoided; a maximum of two attempts is recommended. Supraglottic devices (laryngeal mask, laryngeal tube) are very efficient alternatives in the event of intubation failure and can be handled by non-medical professionals.^(1,9,11)^

Before intubation, two ventilations are recommended for every 30 chest compressions. After intubation, ventilations should be uninterrupted at a rate of 8 to 10 per minute, avoiding hyperventilation.^(9)^ Direct laryngoscopy intubation with 100% O_2_ should be performed with a smaller endotracheal tube. The use of 6 to 7 mm tubes is suggested, or 0.5 to 1.0 mm smaller in internal diameter compared to those used for non-pregnant women. If available, endotracheal tube placement should be verified using capnography.^(1,11)^

Cricoid pressure, intended to prevent aspiration of gastric contents, is no longer recommended, as it offers no benefit and can hinder both intubation and placement of a supraglottic airway.^(13)^

In the second half of pregnancy, the enlarged uterus, elevating the diaphragm, can increase resistance to ventilation. Ventilation should be sufficient to promote visible chest rise, but hyperventilation should be avoided, as it can increase intrathoracic pressure, hindering venous return to the heart and impairing cardiac output during resuscitation.^(8)^

If attempts to establish an airway and mask ventilation are not possible, guidelines for establishing an emergency invasive airway should be followed.^(9)^

### How should chest compressions be performed in cardiopulmonary resuscitation during pregnancy?

High-quality chest compressions are essential in the resuscitation process, promoting the return of spontaneous circulation. They should be performed with the pregnant woman in the supine position. If the uterus is palpable above the umbilicus, it should be manually moved to the left, avoiding aortocaval compression. Lateral tilting of the patient is not recommended, as it reduces the effectiveness of compressions.^(1,8,9)^

The compression rate should be between 100 and 120 per minute, and the chest should be compressed to a depth of 5 to 6 cm with each downward movement. Hands positioned in the center of the chest, over the lower portion of the sternum, without the need for adjustments due to pregnancy. After each compression, the chest should be allowed to fully recoil, avoiding continuous support on the pregnant woman’s torso. Chest compressions should be uninterrupted, except during defibrillation and pulse checks (in the absence of expired CO_2_ monitoring), when indicated. Rescuer rotation is indicated every two minutes of maneuvers or every five cycles of 30:2 chest compressions/ventilations. Although the use of a rigid surface under the chest is indicated, this should not delay the start of resuscitation.^(1,8,9)^

Chest compressions and other procedures may trigger the release of aerosols. Cardiopulmonary resuscitation teams should use appropriate personal protective equipment. In pregnant women with suspected or confirmed COVID-19, influenza, or other similar pathogen infection, the team leader should minimize the number of attending professionals and encourage the use of personal protective equipment. Furthermore, the ventilation devices used must have HEPA (high-efficiency particle air filter) filters, and patients without an invasive or advanced airway in place should have their nose and mouth protected with a towel or similar until an airway is obtained.^(1)^

### How should venous access and aortocaval decompression be provided during cardiopulmonary resuscitation in pregnancy?

In second-half pregnancies, venous access should be established above the diaphragm. Medications administered via the femoral vein may fail to reach the maternal heart due to aortocaval compression, which persists until delivery of the fetus.^(1)^ Obtaining two accesses via the cubital veins using a 14-gauge Jelco/Abocath catheter is as efficient for volume replacement as central accesses, with the disadvantage of not allowing hemodynamic monitoring. If central or cubital venous access is not feasible, rapid intraosseous access can be obtained, also in the upper limb (humeral), with similar efficacy for drug administration. Intraosseous access requires specific equipment (kit).^(14,15)^

Endotracheal drug administration is no longer recommended, as it is associated with unpredictable and generally low plasma concentrations, as well as lower rates of return to spontaneous circulation and survival.^(11)^

In pregnant women with the uterine fundus at or above the level of the umbilicus, manual left uterine displacement is indicated. This maneuver aims to: (1) minimize aortocaval compression; (2) optimize venous return (preload); (3) Maintain the supine position of the upper trunk, preserving the effectiveness of chest compressions and the adequacy of stroke volume.^(1,9)^

**Figure 2:**
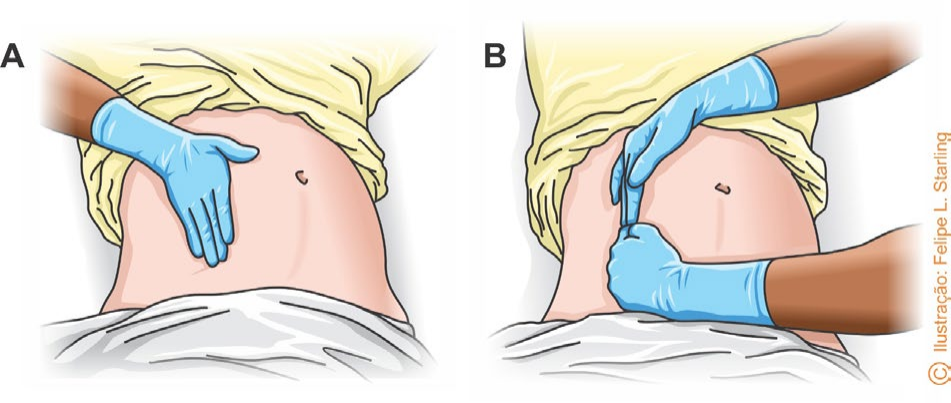
Manual uterine displacement technique with one hand (A) or two hands (B)

Source: Illustration by Felipe Lage Starling (authorized).

To perform the maneuver, one of the rescuers should position one or both hands on the right lateral border of the uterus and displace it laterally to the left, approximately 3 to 4 cm from the midline (Figure 2). Tilting the table to achieve uterine displacement is not indicated, as it impairs chest compression maneuvers, reducing the success of resuscitation. In addition to optimizing chest compressions, manual left uterine displacement facilitates access to the airway and venous access.^(1,9)^

**Figure 3:**
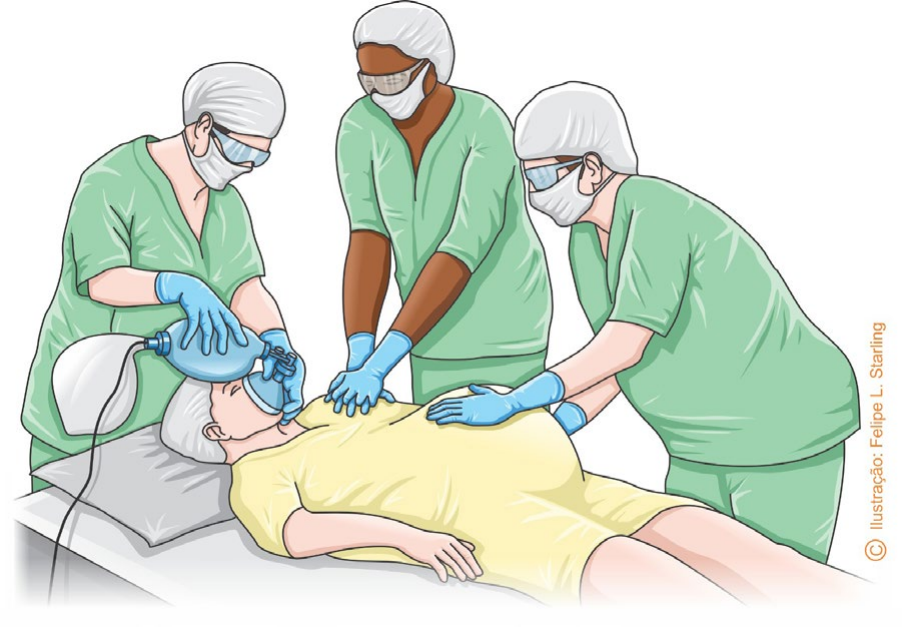
Cardiopulmonary resuscitation in pregnancy performed by three rescuers with responsibilities for ventilation, chest compressions, and manual uterine displacement

Source: Illustration by Felipe Lage Starling (authorized).

Manual uterine displacement necessitates an additional rescuer in the resuscitation of the pregnant woman, besides one responsible for ventilation and another for chest compressions. Ideally, the rescuer who displaces the uterus should monitor the time of CPA and perform resuscitative hysterotomy at the appropriate time (Figure 3).^(1,9)^

### When and how to proceed with defibrillation during cardiopulmonary resuscitation in pregnancy?

Most pregnant women in CPA present non-shockable risks (pulseless electrical activity and asystole). However, cardiac arrests associated with ventricular arrhythmias require defibrillation. Ventricular fibrillation and pulseless ventricular tachycardia are the main rhythms classified as shockable, present in 20% to 30% of pregnant women in CPA, and are frequently associated with hemorrhage, hypoxemia, thromboembolism, toxicity, and electrolyte disturbances. These conditions are potentially reversible with appropriate treatment.^(11,16,17)^

To perform defibrillation, the right defibrillator paddle should be positioned in the supero-anterior portion of the right hemithorax, below the right clavicle, and the left defibrillator paddle in the infero-lateral portion of the left hemithorax, near the boundary between the thorax and the upper abdomen. When positioning the paddles in an adult woman in CPA, it may be acceptable to adjust the position of the bra instead of removing it.^(1)^ Interruption of cardiac massage and ventilation should be minimal. When previously installed, cardiotocography or any other fetal monitor should be removed before the shock. Conversely, pacemakers or implantable cardioverter-defibrillators do not prevent defibrillation, but require that the paddles be placed at least 2.5 cm (1 inch) away from their edges.^(1,2,11)^

The physiological changes of pregnancy do not determine changes in transthoracic impedance or transmyocardial current. Defibrillation protocols for pregnant patients remain identical to those for non-pregnant adults. For biphasic defibrillators, administer an initial shock of 120 to 200 joules, increasing the energy for subsequent shocks, if the first is unsuccessful. If using a monophasic device, a constant energy of 360 joules is recommended.^(1)^

### When and how to determine gestational age, assess fetal well-being, and perform resuscitative hysterotomy in cardiopulmonary resuscitation during pregnancy?

Determining gestational age in the care of a pregnant woman in CPA is essential to define the course of action, although no resuscitation procedure should be delayed to obtain this information. In physiological singleton pregnancies, the uterine fundus is at the level of the umbilicus around the 20^th^ week of gestation. From this gestational age onwards, displacement of the gravid uterus should be performed during resuscitation, and resuscitative hysterotomy is indicated, aiming at aortocaval decompression and return of spontaneous circulation. If the fetus is alive, neonatal survival can be achieved in pregnancies beyond 22-24 weeks. In these cases, the neonatology team should be called in.^(1,9)^

The approximate gestational age can be obtained by consulting prenatal care records, information from a companion, or an obstetric physical examination. The formula “fundal height (“uterus tape”) = gestational age in weeks” can be useful, but it can be falsified in cases of maternal obesity, twin pregnancy, uterine fibroids, and intrauterine growth restriction, and in amniotic fluid volume deviations (oligohydramnios, polyhydramnios). Despite its high reliability in determining gestational age, the ultrasound method is not very applicable during CPR of pregnant women due to logistics and time constraints.^(1,17)^

Assessments of fetal heart rate and/or well-being are not recommended during maternal CPR, as the pregnant woman’s condition should guide care. Deterioration of the maternal condition determines the compromise of fetal health. Therefore, the focus of care should be the reversal of maternal CPA. Previously installed fetal monitors should be removed to avoid interfering with resuscitation maneuvers. If CPR is successful and the pregnant woman regains hemodynamic stability, fetal well-being can be assessed using fetal heart rate monitors, cardiotocography, or ultrasound.^(9)^

Resuscitative hysterotomy should be performed if spontaneous circulation is not restored within four minutes of CPA. Cesarean section should be initiated within four minutes and fetal delivery should be completed within five minutes. In non-shockable rhythms and in pregnant women where the time of CPA is unknown or unwitnessed, surgery should be performed as soon as possible.^(9,18)^ In CPAs with shockable rhythms, surgery should be initiated immediately after unsuccessful defibrillation.^(18)^ A significant proportion of pregnant women in CPA return to spontaneous circulation only after resuscitative hysterotomy. The time interval between CPA and the performance of resuscitative hysterotomy is one of the most important factors in maternal and neonatal survival. In cases of CPA occurring during the expulsive phase with the fetus engaged, operative vaginal delivery using forceps or vacuum extraction will be appropriate, provided that fetal extraction occurs within this time limit.^(9,18)^

Resuscitative hysterotomy is justified by evidence that irreversible brain damage can occur in non-pregnant adults after four to six minutes of anoxia. Pregnant women become anoxic earlier than non-pregnant women due to reduced functional residual capacity. Although the alternative of manual left uterine displacement side seems appropriate, the mechanical effects of the pregnant uterus reduce venous return from the inferior vena cava, obstruct blood flow in the abdominal aorta, and decrease thoracic compliance, contributing to CPR failure. Therefore, with the uterine fundus at or above the level of the maternal umbilicus, cesarean section can contribute to aortocaval decompression and, consequently, improve the effectiveness of resuscitation.^(1)^ Cardiac output peaks immediately following fetal delivery as the empty uterus contracts, causing myometrial blood to be autotransfused into the systemic venous circulation. Furthermore, the contracted uterus elevates the vena cava, increasing venous return and stroke volume.^(19,20)^

The probability of a normal neonatal neurological outcome is significantly higher when resuscitative hysterotomy is completed within five minutes. Absence of neurological deficit has been documented in more than 90% of neonates delivered within the first five minutes of maternal CPA. While maternal and neonatal outcomes decline as the interval between cardiac arrest and delivery increases, favorable survival remains possible beyond the traditional five-minute window. Therefore, resuscitative hysterotomy should be performed even if the five-minute limit has passed, as it may still offer a chance for maternal and neonatal recovery.^(21)^ Other factors that favor neonatal survival include the absence of sustained maternal hypoxia before CPA, good prior fetal oxygenation, efficient CPR, and rapid availability of neonatal intensive care.^(3)^

For a timely performance of the resuscitative hysterotomy, the resuscitation team must plan the procedure as soon as CPA is diagnosed.^(1,9)^ An operating room is not a priority, and surgery should be performed at the scene.^(1,22,23)^ When available, the neonatology team should be quickly activated. If an obstetrician or surgeon is not present at the scene, surgery should be performed by a resuscitation physician. An emergency cesarean section kit should be readily accessible and integrated into the resuscitation carts of maternity wards, as well as those of locations capable of providing care and/or transporting pregnant women. If necessary, the entire procedure can be performed with a scalpel. High-quality chest compressions should be continued throughout the surgical procedure, as well as manual left uterine displacement. Sterile technique (rapid antisepsis), use of sterile material and personal protective equipment are recommended.^(23)^ The procedure should ideally be performed within one minute, and the scalpel and hands are the main resources to be used. The abdominal incision can be vertical midline or low transverse (extended Joel-Cohen). In addition to speed, the vertical midline incision provides adequate uterine exposure, less bleeding and access to the diaphragm, which can be useful for subsequent resuscitation interventions (direct cardiac massage). The hysterotomy should be classic corporeal (vertical, 5 to 7 cm from the uterine fundus). Only the fetus is removed and taken for drying, warming and neonatal resuscitation. Due to maternal hypoperfusion, bleeding is usually minimal. The uterine cavity should be immediately filled with compresses, without removing the placenta, amniotic membranes or umbilical cord. The left uterine deviation is discontinued, and CPR continues with the patient in the supine position. Once maternal cardiovascular stabilization is achieved, placental removal, hysterorrhaphy, and laparorrhaphy should be performed.^(23)^ The technical details of resuscitation hysterotomy lack consensus among specialists and scientific evidence. Some authors recommend aponeurosis suturing as an additional step, in order to prevent abdominal extrusion of intestinal loops during the continuation of resuscitation maneuvers. Others recommend placental removal and hysterorrhaphy, aiming to reduce subsequent blood loss associated with the restoration of hemodynamic stability. Broad-spectrum antibiotic prophylaxis and prophylactic oxytocin (continuous intravenous infusion, 20 mU/min) are routinely recommended. Intravenous bolus injection of oxytocin is contraindicated due to the risk of hypotension, cardiac arrhythmias, cardiovascular collapse, and death.^(13,23)^ In hemorrhage-related CPA, tranexamic acid should also be routinely administered (1 gram intravenous infusion over 10 minutes).^(18)^

### When and which medications should be administered in cardiopulmonary resuscitation during pregnancy?

In CPR in pregnant women, the medications and doses are the same as those used in CPR for non-pregnant patients, as the benefits related to the potential to save lives outweigh the possible or known fetal risks.^(1)^

In pregnant women in CPA with non-shockable rhythms (asystole, pulseless electrical activity), which are the majority, epinephrine is recommended from the start of CPR, at a dose of 1 mg intravenously repeated every three to five minutes. The drug should be administered intravenously. After administration, a vigorous flush with 20 mL of saline solution should be performed to ensure that the medication reaches the central circulation.^(24)^ In CPAs with shockable rhythms (ventricular fibrillation, pulseless ventricular tachycardia) that do not respond to cardiac compressions, defibrillation, and vasopressors, the administration of amiodarone is recommended. The initial dose should be 300 mg, as an intravenous bolus. Subsequently, 150 mg should be administered every five minutes. If amiodarone fails, lidocaine should be used at a dose of 1 to 1.5 mg/kg intravenously, followed by 0.50 to 0.75 mg/kg at 5 to 10 minute intervals.^(11)^

In pregnant women with suspected CPA associated with magnesium sulfate intoxication (pre-eclampsia/eclampsia, fetal neuroprotection), the infusion should be stopped and calcium gluconate (10 to 30 mL at 10%) should be administered intravenously or intraosseously at the start of CPR.^(1,9)^ Calcium chloride (10 mL at 10%, intravenous) can also be used in magnesium intoxication, hypocalcemia, and hyperkalemia.^(18)^

In suspected or confirmed opioid intoxication, resuscitation should be initiated normally and the use of specific antagonists (naloxone) is indicated as quickly as possible. The recommended dose of naloxone in opioid-induced CPA is 0.4 to 2 mg, intravenously, intranasally, or intramuscularly, and may be repeated every two to three minutes if there is no clinical response, until spontaneous breathing returns or the condition improves.^(25,26)^

Sodium bicarbonate should not be routinely used in the resuscitation of pregnant women, as it is associated with worsening maternal acidosis and, consequently, fetal acidosis. However, it can be used in cases of severe hyperkalemia, as well as in overdose of tricyclic antidepressants or other sodium channel blockers.^(26,27)^

In CPA due to cardiotoxicity induced by local anesthetics, lidocaine should not be used. Amiodarone is the drug of choice in severe arrhythmias induced by bupivacaine. In this condition, early administration of 20% lipid emulsion (Intralipid^®^) is also indicated.^(26,28)^ An intravenous bolus of 1.5 mL/kg should be administered, infused over one minute and followed by an infusion of 0.25 mL/kg/minute, until at least 10 minutes after the restoration of circulation. If circulation does not restore after five minutes, a second bolus of 1.5 mL/kg may be administered, followed by an infusion of 0.5 mL/kg/minute. The maximum total cumulative dose should be 10 mL/kg in 30 minutes.^(28)^

### What imaging tests are useful in cardiopulmonary resuscitation of pregnant women?

No test should delay the initiation and continuation of CPR. Point-of-care ultrasound (POCUS) can be used to identify reversible causes of cardiac arrest, such as cardiac tamponade, pulmonary embolism, hypovolemia, and pneumothorax. Chest compressions should not be interrupted to perform the examination.^(16,29)^

Transesophageal echocardiography and limited cardiopulmonary ultrasound can aid in the etiological diagnosis of hemodynamic collapse occurring during labor or delivery. They offer the advantages of speed, ease of transport, and diagnostic reliability. They have high sensitivity in diagnosing pericardial effusion, cardiac tamponade, and ventricular failure. Ultrasound is more practical because it does not require interrupting ventilation if the patient is not yet intubated. Echocardiography, on the other hand, requires esophageal passage of the probe and transducer, which necessitates interrupting ventilation in patients who are not yet intubated. After intubation, the procedure can be performed without interrupting ventilation. In addition to its diagnostic efficacy, the examination is useful for placing venous and arterial cannulas for extracorporeal membrane oxygenation (ECMO), and for inserting an intra-aortic balloon pump.^(30)^

### What are the main sequences of cardiopulmonary resuscitation procedures in pregnancy?

**Figure 4:**
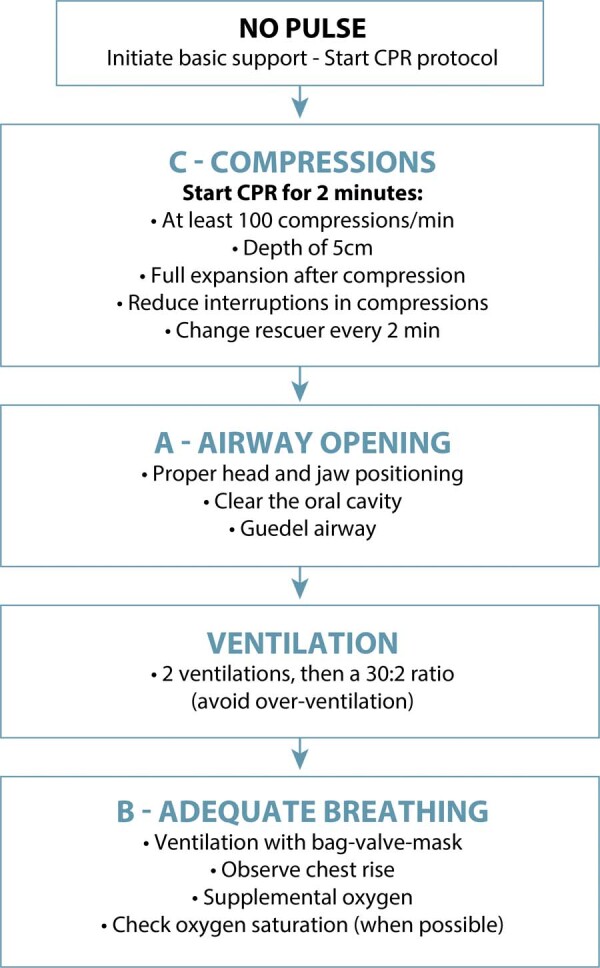
Initial management of cardiopulmonary resuscitation in pregnant women

In cases of CPA in pregnant women, the principles of advanced cardiovascular life support should be implemented immediately, continuing basic support measures, circulation, airway, and breathing. Supradiaphragmatic intravenous or intraosseous access should be obtained, and 1 mg of epinephrine should be administered every 3-5 minutes. Early endotracheal intubation should be performed by the most experienced professional, emphasizing that supraglottic devices (laryngeal mask, laryngeal tube) are efficient and easier alternatives. As with endotracheal intubation, supraglottic devices should also be smaller in size (laryngeal mask numbers 4 or 5, use up to four hours; laryngeal tube numbers 3 or 4, use up to six hours).^(1,9)^Figure 4 presents the sequencing of initial CPR management in pregnant women.

**Figure 5:**
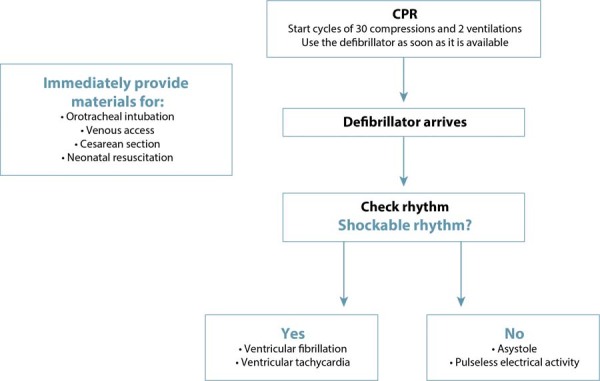
Initial sequencing of cardiopulmonary resuscitation in pregnant women until defibrillator access

**Figure 6:**
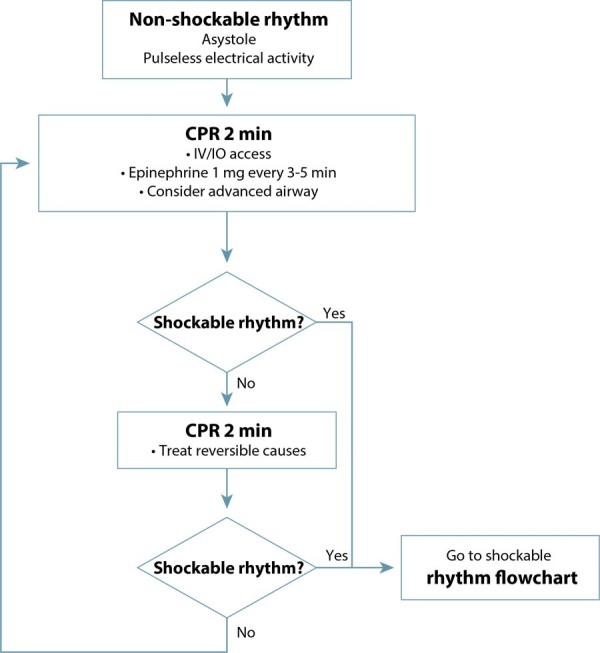
Sequencing of cardiopulmonary resuscitation in pregnant women with non-shockable rhythm

IV: intravenous; IO: intraosseous; min: minute

**Figure 7:**
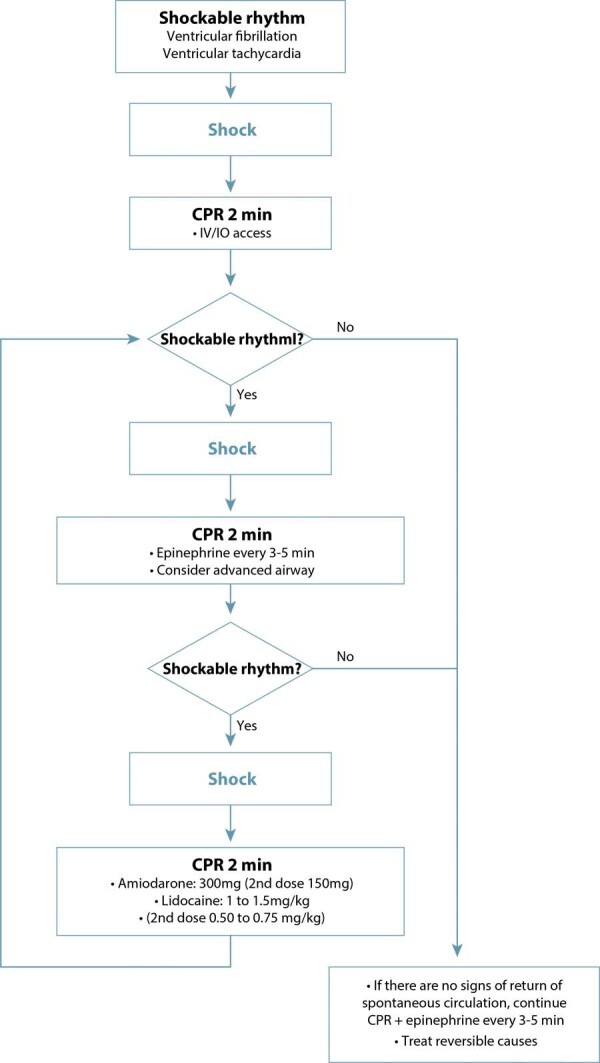
Sequencing of cardiopulmonary resuscitation in pregnant women with shockable rhythms

IV: intravenous; IO: intraosseous; min: minuteIV: intravenous; IO: intraosseous; min: minute

At the beginning of CPR, rapid access to the materials needed for cesarean section and neonatal resuscitation is also essential. Rapid differentiation between shockable and non-shockable rhythms is also necessary, as the resuscitation sequence will be defined according to this diagnosis.^(1,9)^Figure 5 illustrates the initial CPR sequencing until the arrival of the defibrillator. Figures 6 and 7 present the suggested sequences for non-shockable and shockable rhythms. Chart 3 presents the main changes in advanced cardiovascular life support in pregnant women in the in-hospital setting, compared to adults.^(6,31)^

**Chart 3:** Main modifications to advanced cardiovascular life support in pregnant women in the in-hospital setting, compared to adults

.
Call for help (include a multidisciplinary team: anesthesiologist, obstetrician, neonatologist). Manual left uterine displacement using the 1- or 2-handed technique. Earlier advanced airway (prefer a supraglottic device if there is no qualified professional for endotracheal intubation). Use a smaller size tube (0.5 to 1.0 mm smaller than an adult’s). Administer intravenous magnesium sulfate (4 grams) if eclampsia is present. Administer 10% calcium gluconate or 10% calcium chloride intravenously if magnesium sulfate poisoning is present. Administer tranexamic acid (1 gram intravenously) if intrapartum or postpartum hemorrhage is present. Perform resuscitative hysterotomy if gestational age is above 20 weeks or uterus is above the umbilicus (as soon as possible in non-shockable rhythms or unwitnessed CPA; within 5 minutes or after unsuccessful defibrillation in shockable rhythms).

Source: Adapted from Lott et al. (2025).^(^^18^^)^

### What are the appropriate interventions for pregnant women in cardiac arrest who do not respond to standard resuscitation measures?

Some procedures can save the lives of pregnant women in CPA who do not respond to standard CPR. Extracorporeal life support uses a portable pump oxygenator to deliver ECMO in the intensive care unit. There are two types of ECMO: veno-arterial and veno-venous. Both provide respiratory support, but only veno-arterial ECMO provides hemodynamic support. The term extracorporeal cardiopulmonary resuscitation (ECPR) refers to the use of veno-arterial ECMO in conjunction with CPR in patients in CPA. In pregnancy, its best application is in catastrophic cardiopulmonary conditions, such as acute respiratory distress syndrome, heart failure, and cardiac arrest, whether antepartum or in the immediate postpartum period. The need for anticoagulation should not delay or prevent its use, as the benefits appear to outweigh the risks, increasing the possibility of survival in severe cases. The survival rate of pregnant women in CPA treated with ECMO reaches between 55% and 75%.^(1)^ The main difficulties are the availability of equipment and the requirement for specialized multidisciplinary teams, which restricts its presence to large medical centers.^(32)^

Also in locations with availability, the use of intra-aortic balloon pump, cardiopulmonary bypass and ECMO has been described for severe situations unresponsive to resuscitation measures, such as cardiovascular collapse due to amniotic fluid embolism, pulseless electrical activity, anesthetic toxicity, cocaine intoxication and pulmonary embolism.^(32,33)^

In some cases of massive pulmonary embolism, systemic thrombolysis is recommended, despite being associated with severe hemorrhage, the need for invasive therapies (uterine tamponade, laparotomy, hysterectomies) and massive blood transfusion. For these cases, individualized management and discussion with a multidisciplinary team (hematologist, intensivists, and obstetricians) are essential.^(34)^

In the face of a diagnosis or suspicion of amniotic fluid embolism, it is recommended to activate a massive blood transfusion protocol, administer tranexamic acid to control hyperfibrinolysis and, when available, provide veno-arterial ECMO support, aiming at the recovery of cases refractory to clinical management.^(1)^ Inhaled pulmonary vasodilators, such as nitric oxide, may have a role in the management of pulmonary hypertension associated with amniotic fluid embolism, while the use of atropine is not recommended due to lack of benefit and potential adverse effects, such as worsening of the ventilation-perfusion ratio and impairment of diastolic ventricular filling.^(1)^

Pregnant women with ST-segment elevation myocardial infarction (STEMI) may benefit from percutaneous coronary intervention, as the use of fibrinolytics is contraindicated. The condition may be associated with coronary artery dissection, and catheterization of this vessel is necessary for diagnosis and treatment.^(35)^

There is no survival benefit from using direct cardiac massage compared to standard external cardiac massage.^(36)^ This technique may have some advantage in cases of trauma and post-cardiac surgery, when chest compressions are impossible or difficult. In extreme cases, it can be performed via thoracotomy or diaphragmatic access provided by an enlarged vertical incision from a resuscitation cesarean section.^(1)^

Resuscitation should be maintained until the return of spontaneous circulation or its futility is determined. There is no absolute pre-established time, but if there is no return of spontaneous circulation after 20 minutes of high-quality CPR with an advanced airway in place, the probability of success is low.^(37)^ It is recommended to consider extending CPR beyond 20 minutes if the end-tidal carbon dioxide (ETCO₂) is above 20 mmHg, in cases of drug intoxication, reversible causes under treatment (6H and 6T), persistent shockable rhythms, hypothermia or candidates for ECMO/ECPR. The TOR rules recommend considering cessation of efforts when all of the following criteria are present: unwitnessed cardiac arrest, absence of return of spontaneous circulation, no shock administered, and absence of witnessed CPR.^(38)^ Indicators of poor prognosis include unwitnessed CPA, persistently < 10 mmHg ETCO₂ after 20 minutes, initial asystole or agonal rhythm, deep unconsciousness, absence of spontaneous movements/reflexes/breathing, and fixed and dilated pupils, suggesting probable brain death. ETCO₂ should not be used in isolation to decide to discontinue resuscitation, and the final decision should be individualized, based on multiple factors, multidisciplinary when possible, and adequately documented.^(11)^

### How should post-cardiopulmonary resuscitation care be provided during pregnancy?

Once hemodynamic collapse has been reversed and chest compressions are no longer necessary, pregnant women should be positioned in the left lateral decubitus at 90° to avoid aortocaval compression. This recommendation includes patients who have undergone resuscitative hysterotomy.^(9)^ Intensive care with neurological and cardiopulmonary monitoring is indicated.^(39)^

Center temperature lability is associated with increased mortality after hospital CPA. Hyperthermia should be avoided. However, it is not yet defined whether therapeutic hypothermia is beneficial for postpartum women (risk of hemorrhage), as well as for pregnant women who will remain with the fetus in utero.^(9)^

For comatose patients and those who exhibit intentional reactivity after successful CPR, mild to moderate hypothermia, with a target temperature between 32 and 34°C for 24 hours, may be beneficial. In pregnant women with hypothermia, fetal heart rate may exhibit compressed variability and a lower baseline at bradycardia levels. Maternal hemodynamic stabilization is essential for fetal oxygenation, and mean arterial pressure should be maintained above 65 mmHg. Signs of fetal hypoxia and acidosis include the absence of variability and the presence of decelerations in fetal heart rate. The decision to terminate the pregnancy should consider the presence of these signs, maternal surgical risks, and fetal viability (22 to 24 weeks or more).^(40)^

## Final considerations

Cardiopulmonary arrest in pregnancy is associated with high rates of maternal and fetal mortality. The physiological changes of pregnancy, uterine overdistension, and the need for a qualified healthcare team to perform high-quality CPR directly impact the chances of maternal and neonatal survival. Other factors influencing survival chances include the underlying etiology of the CPA, the hypotensive/hypoperfusion status immediately before the arrest, the mother’s location at the time of the arrest, the availability of appropriate resources, the time interval between the arrest and the start of CPR, the timely performance of resuscitative hysterotomy, and the presence of serious comorbidities, such as disseminated intravascular coagulation. The contemporary shift in the age of fertility towards older age groups, the high prevalence of high-risk pregnancies, and the high rates of severe maternal morbidity make CPA in pregnancy the most threatening event to the health of the mother-child dyad, requiring improvements in the availability of appropriate resources for CPR and in the qualification of teams working in obstetric care. Despite the severity of the situation, CPA in pregnant women is rare, which hinders the training and readiness of obstetric teams to provide care. Multiprofessional simulation training is recommended to optimize the quality of care.

## National Specialized Commission on Obstetric Emergencies of the Brazilian Federation of Gynecology and Obstetrics Associations (Febrasgo)

President:

Álvaro Luiz Lage Alves

Members:

Gabriel Costa Osanan
Alexandre Massao Nozaki
Leila Katz
Claudia Margareth Smith
Michelly Nobrega Monteiro
Edmárlei Gonsales Valerio
Eduardo Cordioli
Juliana Silva Barra
Joelcio Francisco Abade
Humberto Sadanobu Hirakawa
Lucas Barbosa da Silva
Sidney Rocha de Mattos Júnior
Roxana Knobel
Daniela Cristina Feliciano Ferreira Nakarato
Juliana Augusta Dias
